# Exploring the occupational therapist’s role in primary health care: Listening to voices of stakeholders

**DOI:** 10.4102/phcfm.v8i1.1139

**Published:** 2016-08-31

**Authors:** Deshini Naidoo, Jacqueline Van Wyk, Robin W. E. Joubert

**Affiliations:** 1 School of Health Sciences, University of KwaZulu-Natal, South Africa; 2 School of Clinical Medicine, University of KwaZulu-Natal, South Africa

## Abstract

**Background:**

Re-engineering of primary healthcare (PHC) was initiated nationally in 2009. There is, however, little information on the role expected of occupational therapists (OTs) in PHC.

**Objectives:**

This research aimed to understand how stakeholders of the Department of Health (DOH) perceived the role of OT in PHC service.

**Method:**

This exploratory, qualitative study used purposive sampling to recruit community health-care workers (CHW; *n* = 23), primary healthcare nurses (PHC; *n* = 5), DOH management (*n* = 5), experienced (*n* = 14) and novice OTs (*n* = 37) who graduated from the University of KwaZulu-Natal. The PHC nurses and the CHW represented PHC clinics in one district in KwaZulu-Natal. Data were collected through semi-structured interviews and focus groups. Interviews with CHWs were conducted in isiZulu. These were transcribed and translated prior to data analysis. Audio recordings of English interviews and focus groups were transcribed. Data for each participant group were inductively and thematically analysed to identify the themes.

**Results:**

The findings provided an indication of the role of OTs in PHC settings. All participants perceived the role of OTs as predominantly curative/rehabilitation-based and individualised. Participants had a limited understanding of the key principles of PHC. They identified a need for adult and paediatric rehabilitation and early childhood intervention. Limited mention was made of population-based approaches, collaborative, and health promotion and prevention programmes.

**Conclusion:**

The study has highlighted that neither management nor OTs seemed to align practice and planning according to PHC principles. A review of the theory and experiential learning in the OT programme is required.

## Introduction

South Africa uses a geographically based district health system to provide public health care.^1^ Service delivery in the health system is offered at tertiary, secondary, district and primary levels of care.^1^ A particular challenge is that therapists are also expected to offer rehabilitation services to communities at community healthcare centres and primary healthcare (PHC) clinics in addition to providing services at a district hospital level.^2,3,4^ In response to the high burden on the public health system, the Department of Health (DOH) has introduced the National Health Insurance (NHI).^5^ A key strategy of the NHI is an emphasis on the delivery of a comprehensive PHC service. The re-engineered PHC package has a strong community focus which emphasises promotion, prevention and rehabilitative services.^5,6,7^ PHC comprises municipal ward outreach teams, district specialists teams and school health teams.^8^ It acknowledges ‘health care as a universal right, encompasses promotion of healthy living, disease prevention, early detection, treatment of illness, community-based disease management and rehabilitation.’^9^ The PHC approach of the Alma-Ata declaration encourages increased consultation with stakeholders in decision-making and for shared responsibility for health amongst individuals, community groups, health professionals and government.^10,11^

The DOH has issued several guidelines such as NHI policy,^5^ National Service Delivery Areas,^12^ KwaZulu-Natal (KZN) strategic plan 2014–2019,^13^ National Rehabilitation Policy,^14^ and Draft Framework and Strategies for Rehabilitation and Disability.^15^ These documents offer broad outlines of services expected from professionals at various levels of the health system. The role of the PHC nurse and doctor is well-described. However, despite numerous policies, position and discussion papers from national and provincial government, non-governmental organisations (NGOs) and health professions organisations (e.g. the Occupational Therapy Association of South Africa), there is a paucity of published papers on the role of the allied PHC professionals and literature related to whether practice adheres to policy.^16,17,18,19,20^

KZN has the second largest population in South Africa (approximately 10.57 million), with 9.2 million people relying on public health care.^13,21^ KZN continues to have the highest burden of HIV and TB in the country. A recent KZN hospital survey (2011) revealed that most patients are admitted to public health-care facilities for infectious diseases (39%); non-communicable conditions (37%) and injury from accidents and violent trauma (23%).^13^ Physical, cognitive and language delays in HIV-positive children have been also reported amongst those admitted.^22,23,24^ Research further suggests that many adults with HIV/TB and chronic non-communicable diseases are likely to require rehabilitation and mental health services.^13,20^ These findings suggest a need for remedial, preventative and rehabilitation services in response to a number of health issues. Service delivery and training of occupational therapists (OTs) need to be aligned with the principles of PHC within the DHS. These principles include understanding of universal and equitable access to health care, facilitating community participation in programmes, intersectoral collaboration, and designing population-based programmes.^9,25,26^ Services should focus on health promotion and prevention, support of district health teams and collaboration among professions.^8,26,27^

The KZN-DOH has a memorandum of understanding with the University of KwaZulu-Natal (UKZN) for the training of health professional graduates to deliver appropriate services in decentralised settings.^13^ New OT graduates, as community service rehabilitation therapists, will spend up to 40% of their work-week in PHC settings (clinics, community based services and schools). The community service policy, however, does not specify the nature of services to be offered in these settings.^28^ This obliges health professional educators to review curricula and to ensure that graduates are able to deliver relevant services in PHC settings. Given the change in delivery of services, increased access and greater emphasis on health promotion, there is a scarcity of information on the exact nature and role of OTs in the PHC.

At UKZN, OT students are introduced to the concepts of PHC and community-based practice during a 4-year programme. Students engage in community services placement in peri-urban or urban areas for 6 weeks in their final year. It is also not clear whether the PHC principles taught are being applied during community service. To inform the process of OT curriculum review, this research was conducted to gain an understanding of the current role expected of OTs at a PHC level by exploring the views of KZN-DOH employees with experience and an interest in rehabilitation and OT services. A better understanding of the expected services would impact positively on the design of the OT curriculum. Thus, the experientially based opinions of relevant stakeholders were sought to inform curriculum review. This paper describes the views of stakeholders on OT services to be delivered and suggestions for the improvement of such services at the PHC level.

## Research methods and design

This study used an exploratory qualitative design to explore how to align occupational therapy education more appropriately with PHC in KZN. The role of occupational therapy at primary health level and the current services offered were the focus of this study and both a rural and peri-urban sites in KZN served as sites. Multiple methods of data gathering (semi-structured interviews, focus groups and field observations) through multiple data sources (DOH stakeholders) ensured triangulation and richness of data about the role of occupational therapy in this province.^29^

### Setting

The study was conducted in KZN as the UKZN-OT academic programme needs to be aligned to KZN-DOH needs in order to train graduates who deliver services that are appropriate for peri-urban and rural hospitals in this setting. The uGu and uMkhanyakude districts have been designated as rural nodes by the KZN DOH.^1^ The hospitals and clinics in uGu are also potential sites for decentralised training for OT training at UKZN and thus made these areas relevant for the study.

The uGu Health District, located in the lower south coast region of KZN, consists of 16% urban and 76% rural areas.^30,31^ uGu has a population of approximately 722,484 people, of which 60% are aged between 15 and 64 and 33% are younger than 15 years.^31^ The unemployment rate is 35%, and the households have an average income per capita of between R800 and R2953 per month. Fifty percent of the households are female headed, 24% have access to piped water inside their homes and 18% have access to flushing toilets.^30^ The district provides health service through the PHC approach and using a District Health System for all levels of care.^31^ UGu has two district and one regional hospital serviced by experienced and novice OT.

uMkhanyakude is a northern district of KZN, with a population of approximately 625,846, living mainly in rural areas.^32,33^ Fifty-five percent of the population is aged between 15 and 64 years, and 40% is less than 15 years old. The unemployment rate is 42%, and 25% completed the senior certificate examination.^32^ Thirteen percent of the households have access to piped water indoors, and only 9% have access to a flushing toilet.^32^ There are four district hospitals in uMkhanyakude which have OT and community service OT, namely Manguzi, Mosvold, Mseleni and Bethesda.^32^

### Participants and sampling

Twenty-three community health-care workers (CHWs) and five PHC nurses were recruited in the uGu district to gather perspectives of DOH employees at a PHC level. Purposive sampling was used to recruit CHWs and PHC nurses who either worked for or visited the eight PHC clinics that were serviced by the community service OT. An isiZulu-speaking resident of the uGu district served as a research assistant. This research assistant visited each of the eight PHC clinics to explain the purpose of the study to the PHC nurses and CHWs and recruited the PHC nurses and CHW.

In addition five key informants were selected from the DOH management (*n* = 5). These informants could provide views on the expectations of OTs at PHC level from managers representing different strata of DOH. A manager involved in PHC and rehabilitation was recruited from each level within the DOH system, that is, to represent views from national, provincial, district and hospital levels and PHC levels.

Fourteen established OTs with experience at DOH and NGOs for more than 5 years from the UGu and uMkhanyakude districts were recruited. Established therapists were recruited from two districts because of the small number of therapists that had met the inclusion criteria in the uGu district and to allow for redundancy during data collection. The established therapists’ were based at district level hospitals but were also required to deliver a service at a PHC level. These therapists’ views therefore offered an opportunity to gain an understanding of the current services offered and their perceptions of the OTs role for the future.

Lastly, UKZN occupational therapy graduates from the 2013, 2014 and 2015 cohorts (*n* = 70) who had completed their community service in KZN were invited to participate via email. Of these, 39 novice occupational therapists (novice OTs) participated in the study. The three cohorts of graduates had all provided services at PHC clinics during their community service. The novice OTs’ perspectives were sought to provide an understanding of the services offered, their perceptions of the role of OTs in a PHC setting and whether they had translated theoretical PHC knowledge into practice.

### Data collection methods

Focus groups and semi-structured interviews were used to collect data from CHWs, PHC nurses, KZN DOH key informants, and the established and novice OTs. The responses were based on the participants own perceptions and experience of working at a PHC level (See Table 1 for Summary). The participants were interviewed telephonically or at a place of their choice for their convenience. The transportation costs incurred by CHW were reimbursed and they received lunch because of the waiting time.

**TABLE 1:** Data collection methods.

Participant–stakeholder group	Data collection methods	Core questions/observations
CHW (*n* = 23) North and south uGu districts	4 focus groups in isiZulu with 4–5 participants, 35–50 minutes in duration 1 semi-structured interview in isiZulu, 30–45 minutes in duration Conducted by research assistant	Current services at the PHC service delivery level Common difficulties experienced by people with disability and chronic illnesses in their community Perceptions on the role of OT in PHC.
PHC nurses (*n* = 5) North and south uGu districts	5 semi-structured interviews in English, 30–45 minutes in duration Conducted by the principal researcher	Current services at the PHC service delivery level Common difficulties experienced by people with disability and chronic illnesses in their community Perceptions on the role of OT in PHC.
DOH managers (*n* = 5) One national manager Two KZN provincial One regional hospital One uGu PHC manager	5 semi-structured interviews in English, 45–60 minutes in duration Conducted by the principal researcher	Current services expected at a PHC service delivery level Perceptions on the role of OT in PHC
Novice OTs (*n* = 39) UKZN graduates 2013, 2014, 2015	Twenty-nine semi-structured interviews in English 40–50 minutes 1 focus Group in English with 10 participants, 45–60 minutes in duration to follow up on themes emerging from the interviews. Conducted by the principal researcher	Current services offered at PHC level, Understanding of PHC Perceptions on the role of OT in PHC
Established OTs (*n* = 14) uGu and uMkhanyakude districts	14 semi-structured interviews in English, 45–60 minutes in duration. Conducted by the principal researcher Therapists from two districts sought because of limited numbers that met the inclusion criteria in uGu and to achieve data redundancy	Current services offered at PHC level Understanding of PHC Perceptions on the role of OT in PHC
Principal researcher	2 participant observations In each of these, the researcher followed the novice occupational therapist for the day while they were delivering a service at a PHC clinic and conducting home visits	Current services offered at PHC level Where the service is provided and challenges experienced Therapists focus of intervention, interactions with multidisciplinary approach to therapy (MDT) and the expectations of the patients who come into the clinic or where at home visits

*Source*: Authors own work

CHW, community health-care workers; PHC, primary health care; DOH, department of health; OT, occupational therapist

### Data analysis and trustworthiness

The audio-recorded isiZulu data were transcribed and translated. All the English-audio-recorded data were transcribed verbatim and checked for accuracy against the audio recordings. The data were read and re-read to identity the first level of coding using inductive data analysis.^17^ Second level of analysis involved comparison of the themes from the various data sources.

Trustworthiness of the study was obtained through several methods to ensure that rigour was used and richness obtained from the data collected: (1) data source triangulation was used through interviewing members of the different cadres of DOH employees (2) data method triangulation was ensured through using different data collection methods such as interviews and focus groups, observations, field notes and participant observations carried out with the community OT and community healthcare worker; and finally (3) the researcher wrote a reflexive statement prior to commencing the study to bracket potential bias,^21^ that is, the researcher is an OT and staff member at UKZN.

### Ethical considerations

Ethical approval was obtained from the Biomedical Research and Ethics Committee of the UKZN (BE248/14). Permission for the study was obtained from the district managers at the various sites and the KZN Provincial ethics committee. All participants signed a consent form and were informed of their rights.

## Results

Two main themes emerged from the data, that is: perceptions on the role of OTs in PHC and recommendations to improve OT service delivery in PHC. The views of the five participant groups were represented under each theme and substantiated by relevant quotes. The CHW and PHC nurse participant group had interacted with members of the community regularly and were based at PHC clinics visited by community service OTs which provided them with insight into OT services required at this level. The DOH manager participant group provided insight into DOH expectations for OT services from a policy and administrative level. And finally the OT participant group (novice therapist and established OTs) articulated their views of services based on their own particular experiential knowledge and their professional expertise.

### Role of occupational therapist in primary health care

The participant views of the role of OT in PHC related to two subthemes:

services for adults;

and services for early childhood intervention.

### Services for adults:

#### Primary healthcare nurses and community healthcare workers

The PHC nurses and CHW groups initially had difficulty with describing the role of an OT. Both groups were able to answer questions around the role of therapy in PHC. The participants’ views indicated confusion between the roles of OTs, physiotherapy and other therapists.

Both participant groups voiced the need for multidisciplinary teamwork to improve the occupational engagement of clients with disabilities. They emphasised the need to include nurses and CHWs in interventions. They recommended that interventions should include caregiver training with families and assisting people with disabilities (PWD) in performing basic occupational tasks such as bathing and household chores. They also need training to access and use assistive devices. The PHC nurse group expressed a need for therapists to assist young adults to cope with stress and improve their mental health.

‘it’s a service that is rendered to people to encourage them to live positively where they have disabilities, help them to do things and help them with some handwork.’ (Semi-structured interview 2, PHC Nurse)

‘Younger people have got problems, like psychological problems like they come in with overdoses because of certain problems, maybe about the boyfriend at home, others are staying with stepmothers who are worrying them.’ (Semi-structured interview 1, PHC Nurse)

Both these groups thought that therapists should provide health promotion programmes. They requested that education programmes be offered to understand and demystify different diagnoses in the community. They also asked for sessions for CHW’s, nurses and the role of therapy, potential exercises, and methods to assist PWDs. The CHW participant group expressed concerns about being poorly informed and therefore unprepared to assist PWDs. Both the CHWs and PHC nurse groups expressed a need for OTs to conduct home- and follow up visits on PWDs.

‘when you do household visits we find problems for example if we come across someone who’s paralysed, we don’t know how to approach them so they can feel better. We need more training.’ (CHW focus group 1)

‘therapist must do home visits and follow ups on their patients to see if they are improving on their conditioning or worsening.’ (Semi-structured interview 3, PHC nurse)

The CHW group suggested a need for OTs to initiate skills training for income generating projects and training in options on alternative vocations for PWD with the potential for employment. OTs should initiate support groups for PWDs and their caregivers for opportunities to socialise, spaces to share their emotions and to promote health education sessions in the community.

‘you find that some are young but have arthritis and cannot walk properly but they need to do something like computers’ or something.’ (CHW focus group 1)

‘if they (OT) can create support groups for people with disabilities there would be progress.’ (CHW focus group 2)

### Department of Health management participant group

The following OT service needs emerged from this group:

All participants noted that therapy should be aligned to DOH health needs, that is, a focus on non-communicable diseases, sequelae of HIV/TB, violence and trauma and rehabilitation service in the community. Rehabilitation should include mobility and orientation instruction in the community, retraining for employment, provision of assistive devices and reintegration into the community. Additionally, services for people with mental health disorders and follow up with people diagnosed with substance abuse (especially post hospitalisation) were recommended. All the participants voiced the need for prevention of disability or early detection. This would include collaborating with the CHWs and school health teams to screen for individuals with, or at risk of physical or mental disability. The national and provincial participants emphasised the need for secondary prevention strategies, for example, caregiver training with bed mobility and positioning to prevent pressure sores and contractures. Furthermore health promotion and primary prevention programmes were needed. These could include engaging with youth at school to promote healthy living and prevent drug abuse, teenage pregnancies and engaging with adults to reduce complications from non-communicable diseases. This group thought that OTs at Community health centre (CHC) and district level should also provide a service at a PHC clinic level as part of their job.

‘…we’ve got to prevent disabilities, we’ve got to identify early and intervene early… we are losing a lot of those who are referred from hospitals back into communities, we are not reintegrating our clients back into the community, and family adjustments and all of those things.’ (Semi-structured interview, DOH manager provincial level)

‘OT’s are best placed to do vocational rehabilitation, also not forgetting mental health to give people tools to deal with stressful situations etc.’ (Semi-structured interview, DOH manager national level)

### Established occupational therapists and novice occupational therapist participant group

The OT participant group voiced that services focussed predominantly on rehabilitation with some health promotion. According to this group, their main services at PHC level included individualised rehabilitation of patients with cerebrovascular accidents, hand conditions, arthritis, TB/HIV sequelae, and chronic non- communicable diseases and provision of assistive devices. Health promotion services were conducted either around health education at PHC clinics and awareness campaigns in the hospital. The OT participant groups suggested group interventions for clients to gain insight and support during group education sessions. Only a few participants offered programmes for people with mental health disorders. Additionally, the OTs identified a need for programmes to assist people with substance abuse (alcohol and marijuana).

The peri-urban and rural OTs had different perceptions regarding rehabilitation. The rural OTs articulated the need for collaboration with community stakeholders and home based visits for rehabilitation to improve access to services. The peri-urban OTs focussed on hospital and clinic based services. The OT group emphasised the need for shared goals with the client and family during intervention planning and empowering clients through improved awareness of their rights. There was no mention of prevention programmes or screening.

‘You have to visit their [*PWD*] home, teach alternative methods to the patient be more productive, educate on how to manage which will cover the empowering aspect of the individual and the immediate community.’ (Semi-structured interview 10, established OT)

‘Resources within in the community I am still finding it a bit hard because it is not the same as varsity. We already had those networks given to us, we were connected with these people [*stakeholders*] by the time we got there [*service learning rotation*]. Now trying to find, even find the person that I am supposed to connect with like I don’t know.’ (Semi-structured interview 4, novice OT)

### Services for early childhood intervention

#### Primary health care nurses and CHWs participant group

This group did not mention a role for OT in ECI. The CHW participant group felt that OTs should assist with identification and facilitation of schooling placements.

#### Department of Health manager participant group

DOH manager participant group stated that OT had a role in the early detection of disability and providing intervention for children especially the babies identified through the DOH Hypoxic Ischaemic Encephalopathy (HIE) and high risk baby screening programme. An additional need to provide interventions for children with HIV/TB and or developmental delays resulting from malnutrition and HIV/TB was also identified. Which also included early stimulation programmes, education with mothers and caregivers and environmental redesign of playgrounds and crèches.

‘OT’s should work in early childhood intervention which would cover a number of aspects, you know like to detect disabilities in infants and work in World Baby clinics to help nurses screen for certain conditions, to be able to work at Early childhood development centres for children. OT’s can play a role in fostering development of children who are born prematurely and educate mothers so that they know why they do what they are taught to do.’ (Semi-structured interview, with DOH manager national)

#### Established occupational therapist and novice occupational therapists (occupational therapist participant group)

This group expressed the importance of OT being involved in early childhood intervention especially with the HIE, high risk babies and children born with congenital abnormities. Screening of children with disabilities and developmental delays could occur when babies visit the PHC clinic for their immunisation was suggested. Another role they suggested was to empower teachers to identify children with learning disabilities and to share strategies with teachers to assist such learners. The established OT participant group voiced a need to assist families’ with the psychological acceptance of the child having a disability and that such parents may benefit from having a safe space to share their feelings, a place to ask questions and where mothers/caregivers can be educated on how to provide stimulation to promote general development. A need for introducing adolescence programmes which would promote healthy lifestyles and facilitate their coping better with stress and anxiety was expressed by this group.

‘we need to work with children with developmental delays, cerebral palsy, we’ve started a group because of the high number of children. We find kids who have been going to the PHC clinic for their immunisation but not identified as having a problem. I feel that if we go to the PHC clinic, that’s when we actually can see the children and can start working with them at an early age.’ (Semi-structured interview 6, novice OT)

‘we have to start working with the family or the parents to get them on a level to be willing to or to be ready to also make changes in the child’s life because they have to deal with all their broken expectations.’ (Semi-structured interview 4, established OT)

### Recommendations for the occupational therapist service in primary health care

#### Primary health care nurses and CHW participant group

The recommendations revolved around service delivery, multi-disciplinary team work and individualised therapy. Both groups felt that the OTs should be employed at a clinic level. They should collaborate with PHC nurses and CHWs to facilitate an improved understanding of the profession and awareness of the information given to the patients and their families. The PHC nurse group expressed the need for improved communication and the CHW participant group expressed a need for the OTs to be able to speak to the patients in their own language. Additionally, it was suggested that OTs should have set dates with the CHWs to facilitate home visits.

‘there should be nurse at the side of the OT. Whereas now we find that we just show them the venues they are supposed to work in. If the nurse was there she would know exactly what the therapist is telling relatives or the person with a disability, so that the nurse can take up from there and do a follow up.’ (Semi-structured interview, PHC nurse 4)

‘It would help if [occupational therapists] could come closer to the places we work in, we would be able to work together and improve the interaction.’ (CHW focus group 2)

#### Department of Health managers’ participant group

The DOH manager participant group suggested that the PHC service needs should be aligned to the DOH policy and the negotiated service delivery areas (NSDAs). For example OTs should link with the PHC clinics and the integrated school health programme for ECI. There needs to be more emphasis on health promotion especially healthy lifestyle and collaboration with community stakeholders. OTs need to work with patients with non-communicable diseases to prevent disability. The need to change to a population-based approach but no examples of how they envisioned OTs to do this was mentioned.

‘Nobody is talking about promotion of health in the perspective of a therapist, nobody is talking to health promotion and prevention of disabilities and diseases that is the primary care for me which is missing.’ (Semi-structured interview, DOH manager Provincial)

‘When you enter into a community; you need to understand the system and the structures that are there. If you go into an urban community, you will find the civic organisations, the municipal counsellors and all that. So you do need to be able to collaborate with all the stakeholders.’ (Semi-structured interview, National Manager)

#### Established and novice occupational therapists participant group

Recommendations focussed on rehabilitation and individualised therapy. This group recommended that services should be planned around the burden of disease in the area to be serviced. This group voiced the need for stronger collaboration with the CHWs. Some of the suggestions included having CHWs present at therapy sessions and on teams responsible for the home visits to promote carry-over and continuity of the therapy.

It was suggested that a database be developed of all the adult and paediatric patients referred from district hospital to PHC clinics to prevent ‘lost referrals’. This would strengthen the referral pathway and allow for better follow up. It was voiced that if PWDs had access to early therapy that it could prevent secondary complications and re-admission to hospital. Lastly, the OTs articulated the need to negotiate goals with patients and their families and use a problem solving approach to therapy. There was no mention of how population-based intervention, group intervention or prevention programme could be implemented.

‘You need to get the people who you are working with involved. An example sometimes you get a person who is blind who wants help with occupational mobility. You would think of teaching him to get on a taxi or going to the shops but he might want to get out of his house to sit under the tree in the shade. I would never have come up with that goal but for this person it would change his life because that’s what the family do in the afternoon, they sit under the tree and talk.’ (Semi-structured interview 5, established OT)

## Discussion

The findings of the study revealed that current-role players perceived the role of OTs predominantly as being impairment and strongly rehabilitative focussed. Intervention is predominantly individualised with limited group (paediatric intervention) and no population based programmes. The findings indicate a lack of universal programmes to promote broad access to interventions, limited community participation and intersectoral or multi-sectoral collaboration. Current practices lacked the focus on health promotion and disease prevention, including secondary and tertiary prevention, and interprofessional collaboration was limited. The role of OTs in population–based programmes and in preventative- and promotive interventions were also unclear. These findings implies that OT services at current PHC public health facilities may not meet the norms as stipulated for a comprehensive PHC approach, but that the strong medical model is still being followed.

The current role of OTs in rehabilitation of adults was clearly articulated. It was voiced that OTs needed to provide caregiver training, especially for PWD and that OTs needed to institute support groups for PWD and their caregivers to voice their needs, share ideas regarding adapting to a disability and how to regain control over one’s health.^34,35,36,37,38,39^ The CHWs and DOH managers viewed vocational skills retraining as an important role for OTs to retrain patients for productive roles in their communities. This is particularly relevant given the high unemployment rate in both the uGu and uMkhanyakude districts. All participants agreed that OTs needed to provide services to patients with mental health disorders services. OT interventions should include community education both in the workplace and that of teachers to screen for learning disabilities.^25,26,27,28,29,30,31^ Support groups for adults could be started at PHC clinics to cater for addicts of substance abuse. DOH managers also saw a need for OTs to provide orientation and mobility services for people with limited vision or blindness, especially given the absence of sufficient services.

The findings have indicated that current OT services offered to adults at PHC are predominantly individualised and only partially aligned with OTASA’s position statement.^17^ Services mentioned are in keeping with aspects of the rehabilitation polices.^14,15^ OTASA endorses a comprehensive population-based PHC approach which includes the core principles of Community-Based Rehabilitation (CBR), human rights-based and multidisciplinary approach (MDT) to therapy.^17^ Furthermore, the fundamental philosophy of OT advocates for health and well-being to be linked through participation in everyday occupations and social inclusion within the family and community.^17,18,19^ Our findings indicate that OTs have a role to play in curative aspects of rehabilitation. However, current practices lacked empowerment strategies, community participation and intersectoral collaboration aimed at equalising and promoting opportunities and social inclusion of PWDs.^9,26,40^ Additionally, there is limited multidisciplinary teamwork and task shifting to the CHWs which is key to improving the access to services to a greater portion of the population.^25,38,40,41,42,43^ Of concern was the observation that current OT practice seemingly lacked the inclusion of members from communities in programme-planning and consultation about health needs. This is disconcerting given the inclusion of this topic in the current undergraduate curriculum.

OT services at a PHC level should be planned around the health burden of the ward/district and health promotion programmes are to be negotiated with the communities to be served.^10,27,44^ Additionally, the focus of intervention should be on enabling members, especially PWDs to engage in activities that promote and improve social reintegration and quality of life. The World Health Organisation’s International Classification of Functioning (ICF) resonates with this sentiment.^45^ The ICF further emphasises the need for therapists to consider contextual, environmental factors and social determinants of health when planning socially inclusive interventions. OT literature recognises the influence of the social determinants of health on the wellbeing of the community^17,39,46,47^ and the need for a population-based approaches to health care.^46^ However, only established therapists based in rural areas reflected on the impact of social determinants on patients’ engagement in daily tasks and their re-integration into the community. There was limited mention of how multi-sectoral collaboration and community participation could aid the well-being and overcoming social determinants to improve PWDs ability to engage with tasks that are meaningful to them. OTs have a unique role in helping PWDs to solve problems of addressing environmental barriers and engage in meaningful tasks that are appropriate to their context, culture and financial constraints. Given the environmental and economic barriers of rural living in the uGu and uMkhanyakude districts, it would be essential that therapists consider these barriers during intervention planning. Such consideration would demonstrate the desirable shift to embrace the social disability model of therapy.^26,34,35,36,38,48^ The approach will assist in retraining and reorientation to implement the principles of PHC in OT services. This would facilitate a more community-based focus rather than the limited clinic-based service which are currently offered. Another concern was that no mention had been made of population-based or universal programmes to increase access to services.

Programmes should address the quadruple burden of disease such as HIV/AIDS, TB, and non-communicable diseases.^39^ Such programmes should include impairment screening and educational programmes at a community level as part of primary prevention and promotive programmes.^39,44,49^ Intervention that addresses primary, secondary and tertiary prevention is essential at PHC level. Primary prevention aims to prevent onset of a condition, secondary prevention aims to prevent advancement and complications post -illness, and tertiary prevention aims to enable a healthy life in the presence of the condition or impairment.^39,50,51,52^ Sherry^39^ gives the example of providing diabetic foot care to those patients at risk for amputations as part of secondary prevention programme and providing ideas to support people with a disabilities at home to promote participation and reduce the impact of the disability as part of a tertiary prevention programme.^39^ It is of concern that only the DOH participants mentioned prevention programmes, which indicates that these may not currently being offered by OTs.

The role of OTs in PHC in South Africa is not new. It is however necessary to elaborate on the expected role within the newly re-engineered PHC context. Despite agreement to offer health promotion and prevention, community empowerment and CBR, participants in this study had limited vision for services beyond engagement in education sessions at the clinics and support groups for PWDs. The lack of clarity on the role of OTs in health promotion and prevention may be one of the reasons why OTs services remain traditional and hospital focussed. OTs need to be aware of the danger of transferring their approach to therapy from hospital care to a new setting (PHC clinic) without adequate consideration to prevention and health promotion as part of their services.^44, 46,47,49,50,51^

OTs have to play a role in disability prevention and health promotion in paediatric clients. Participants highlighted their particular role in early detection of disability, developmental delays and provision of intervention in high risk babies, HIE babies and malnourished children. The possibility to screen children for developmental delays during immunisation visits to the clinic is a proactive suggestion. The need to provide education to mothers and caregivers on parenting, early stimulation for normal growth and development should be included. A need was identified for OTs to assist parents and the community to accept having a child with a disability. Collaboration with appropriate role players to assist with community programmes to promote cognitive, physical and social development would be another role and is in keeping the KZN DOH’s strategic plan to promote child and maternal health and wellbeing.^13^

OT should be working in schools and with teachers to identify and assist leaners with learning disabilities. They could also redesign playgrounds to stimulate physical and mental development of children. It was suggested that OTs could promote wellbeing in adolescence by helping them to develop life skills to discourage self-harm and behaviours which threaten their health. The example was noted of how the increased teenage pregnancies and substance abuse amongst adolescents could be prevented if OTs advise communities on sports, leisure and recreational programmes to build positive and affirming behaviours. OTs could thus be part of school health teams which is one of the three streams of PHC.^8,27^ OTs providing a service to children and adolescence is particularly relevant given that 40% of the population in uMkhanyakude and 33% of the population in UGu is under 15 years old.^30,32^

OTs need to focus on the health of the family and community. This will need exploration of how to adapt their roles to ensure that interventions are population focussed, collaborative, evidence-based and outcome-oriented to addresses the burden of disease, for example, task shifting within a MDT, multi-sectoral collaboration or offering a service as a consultant rather than one on one intervention.^1,3,44,48,49,50,^ OTs’ role in improving integrated services at PHC level needs to include community programmes to address substance abuse and educational challenges, teaching life skills, assisting with constructive leisure and social programmes and possibly providing support in learning where there is poor support for learning due to the context of the community. There is a need create a MDT approach with a broad functional approach to intervention.^4,41,42,43^ Stronger links between OTs, CHWs and other cadres of health professionals is essential in order to ensure carryover of therapy and better referral systems to rehabilitation service. This would necessitate the upskilling and task shifting of CHWs.^4,41,42,43,49^ Thus OTs would need to train CHWs in for example, disability related diagnosis, the role of therapy ways to assist people with disability such as correct positioning and processes for accessing assistive devices. The need to train CHWs and Community Rehabilitation Facilitators (CRFs) to facilitate carry-over of intervention and the process of empowerment and enabling the community to make their own decisions is supported by literature.^34,42,43,46^ Empowering these categories of health worker to take over a more prominent role would also help to accommodate the shortage of rehabilitation professionals in these areas. Cultural sensitivity was expressed as an essential part of working in a PHC setting and would include the ability to communicate adequately in the language of the majority of people served within a particular community; this would also help to decrease the power dynamic between therapists and patients, and would enhance reciprocal planning and goal setting.^35,38,41,48,52^

## Conclusion

This paper described some insight into the roles and services required from OTs at a PHC level in the current Public Health System, particularly from the viewpoints of different health professional stakeholders. The study has highlighted that neither management, nor OTs seemed to align practice and planning according to PHC principles. There is a need for a more decisive shift from the narrow medical model with its focus on the individual and his/her impairment to a more social model that considers the social determinates of health, the rights and views of patients and the community. Additionally, there is a need for multi-disciplinary and multi-sectoral planning. The disciplinary silo only promotes isolation and a narrow technical focus rather than the envisioned population-based approach. Since the demise of apartheid, the new health system has put pressure on OT to adapt to new health-care policies and the PHC approach. It is our opinion that the process has been too slow and that OT training needs a greater emphasis on OT interventions within community and PHC settings.

The health professional graduate is a product of higher education programmes. Higher education institutions need to review their curricula to ensure that their graduates are equipped to deliver services aligned to the PHC approach. This qualitative study focussed on the uGu and uMkhanyakude districts only. A limitation was that the views of community members had not been included in this study, a fact which may limited the generalisability of the findings. However, we believe that the findings still provides valuable information to inform the curriculum review within occupational therapy and also other rehabilitation related professions.
